# Optimizing Sensorless Control in PMSM Based on the SOGIFO-X Flux Observer Algorithm

**DOI:** 10.3390/s24030817

**Published:** 2024-01-26

**Authors:** Rencheng Jin, Ji Chen, Peihao Hu, Jianzhang Li

**Affiliations:** Key Laboratory for Micro/Nano Technology and System of Liaoning Province, Dalian University of Technology, Dalian 116024, China; chenji@mail.dlut.edu.cn (J.C.); 22204093@mail.dlut.edu.cn (P.H.); lijianzhang@mail.dlut.edu.cn (J.L.)

**Keywords:** permanent magnet synchronous motor, nonlinear flux observer, second-order generalized integrator flux observer extend, DC component, harmonics

## Abstract

In the realm of sensorless control for a permanent magnet synchronous motor (PMSM), the flux observer algorithm is widely recognized. However, the estimation accuracy of rotor position is adversely impacted by the interference from DC bias and high-order harmonics. To address these issues, an advanced flux observation method, second-order generalized integrator flux observer extend (SOGIFO-X), is introduced in this paper. The study begins with a theoretical analysis to establish the relationship between flux observation error and rotor position error. The SOGIFO-X method, developed in this study, is compared with traditional methods such as the Low Pass Filter (LPF) and second-order generalized integrator flux observer (SOGIFO), employing mathematical rigor and Bode plot analysis. The emphasis is on the methodology and the general performance improvements SOGIFO-X offers over conventional methods. Simulations and experiments were conducted to assess the impact of SOGIFO-X on the steady-state and dynamic performances of sensorless control. Findings indicate that SOGIFO-X demonstrates significant enhancements in terms of reducing the reduced flux observation error, contributing to the advancement of position estimation accuracy and sensorless motor control technology.

## 1. Introduction

Permanent magnet synchronous motors (PMSM) are noted for their robust starting torque, long lifespan, wide speed regulation, and high power factor [1,2]. Widely used across various sectors, they power everything from home appliances to medical devices, transport systems, and industrial machines. Field-oriented control, also known as vector control, is the dominant method for PMSM [3]. This method crucially depends on real-time data regarding the motor’s position and velocity. However, the traditional position sensor has disadvantages of limited use environment and high cost. Addressing the limitations of sensor-reliant systems, the field of sensorless control technology has gained prominence as a vital research area in recent years [4,5,6].

Currently, sensorless control in motors predominantly falls into two categories. The first is the high-frequency injection method [7], which leverages the motor’s saliency effect and is typically applied in low-speed scenarios. The second category is the back EMF method, which includes various control techniques like sliding mode observers [8], Luenberger observers [9], and extended Kalman filters [10], mainly effective in medium- to high-speed ranges. However, at lower speeds, these methods can be negatively impacted by noise and non-linear factors, adversely affecting motor performance.

Position observers based on rotor flux have also been investigated. In sensorless PMSM control, rotor flux observation is key for estimating rotor position and speed, and is especially effective at medium to high speeds [11,12]. Lee et al. [13] innovated a flux observer that precisely gauges rotor position by meticulously analyzing rotor flux. This observer integrates the stator’s electromotive force using a pure integrator, facilitating accurate stator flux computation. It further accounts for voltage drops across both cross- and direct-axis synchronous inductances, ensuring precise rotor flux determination. Chen et al. [14] highlighted that the integral component of flux observers is prone to amplifying sampling errors and DC bias, leading to significant inaccuracies in rotor flux position estimation. Therefore, enhancing flux estimation accuracy is critical for the broader applicability and effectiveness of flux observers in sensorless control.

Building on this understanding, several researchers have proposed innovative solutions to overcome the limitations of traditional flux observers. Holtz et al. [15] developed a method averaging flux amplitude peaks and troughs within a sinusoidal cycle, effectively nullifying the DC bias in flux. Andreescu et al. [16] and Yu et al. [17] introduced a closed-loop phase-locked loop flux observer to address the flux chain error derived from flux estimation and integration, thereby mitigating the errors induced by integral zero drift. However, this method shows limited efficacy in suppressing higher-order harmonic disturbances. Jianhua et al. [18] developed an online flux amplitude correction based on flux chain discrepancies, enabling real-time adjustments to the permanent magnet flux, thus refining its amplitude accuracy. However, this technique is predominantly applied in motor model parameterization. Hinkkanen et al. [19] employed parameter identification for rotor flux observation, which is not amendable to offline corrections. Feng et al. [20] and Zhiqin et al. [21] proposed using a low pass filter (LPF) as a substitute for the integral component in traditional flux linkage systems. This approach effectively reduces DC bias and harmonic distortions, though it requires additional adjustments in amplitude and phase. Xu et al. [22] utilized an SABPF to eradicate both the DC element and high-frequency harmonics in the estimated equivalent rotor flux. Liu et al. [23] developed a flux observer using a hybrid voltage-current model, integrating a LPF with a HPF. However, this approach requires additional phase compensation to function effectively.

Known for its excellent filtering, orthogonal signal generation, and phase extraction capabilities, the Second-Order Generalized Integrator (SOGI) is highly effective in achieving a 90° phase shift in grid voltage signals. Additionally, it efficiently filters higher-order harmonics, making it invaluable for grid applications [24,25,26]. Zhaoping et al. [27] utilized a SOGI within a sliding mode observer to filter back electromotive force, aiming to minimize the high-frequency oscillations in sliding mode. This integration notably improved the system’s dynamic performance and steady-state accuracy. Zhao et al. [28] and Wang et al. [29] developed a multi-SOGI-FLL flux estimator for induction motors, adept at estimating rotor flux while efficiently reducing both DC and higher-order components, albeit with some limitations in DC bias elimination. Xu et al. [30] introduced a fourth-order generalized integrative flux observer, excelling in removing DC bias and harmonics but at the cost of increased computational demands. Jiang et al. [31] introduced a sophisticated third-order generalized integral flux observer (TOGIFO), markedly improving rotor estimation accuracy. However, this method necessitates extensive parameter tuning. 

Despite these advances, many challenges remain in the field of flux estimation. Most of the existing methods cannot completely eliminate DC offset, the filtering effect of high-order harmonics is limited or the amount of calculation is large, and the practicability is relatively weak. In order to solve this problem, based on the existing research, an advanced second-order generalized integrator flux observer named Extend (SOGIFO-X) is developed, which focuses on eliminating the DC bias in the estimated flux and weakening higher-order harmonics. Theoretical analyses affirm the effectiveness of SOGIFO-X in minimizing DC bias and harmonics, thereby substantially improving flux estimation accuracy and the determination of rotor position and speed, while maintaining robust dynamic performance. The SOGIFO-X algorithm’s performance underwent rigorous validation via extensive numerical simulations and empirical testing.

The structure of this paper is organized as follows: Section 2 explores the conventional sensorless PMSM control algorithm using rotor flux observation, offering a concise analysis of how flux estimation accuracy affects rotor position. Section 3 evaluates the steady-state efficacy of rotor flux observers employing first-order integrator, LPF, and Second-Order Generalized Integrator Flux Observer (SOGIFO) by using comprehensive theoretical analysis, and introduces the enhanced SOGIFO-X. Section 4 details findings from extensive numerical simulations and empirical experiments. Finally, Section 5 summarizes the key conclusions of this paper.

## 2. Traditional PMSM Flux Observe

**Figure 1:**
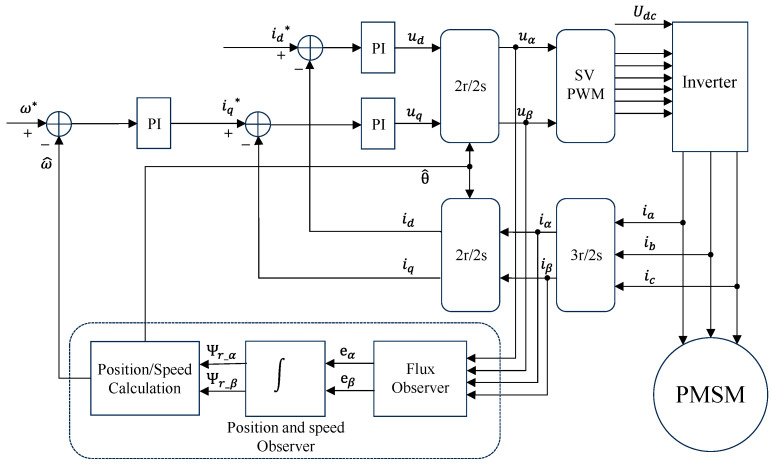
Closed-loop vector control block diagram. * represents the reference value.

Vector control technology is extensively employed in the control of PMSM due to its effective torque control. Figure 1 provides a detailed schematic of vector control, with a special emphasis on the flux observer depicted in the figure’s dashed area. This observer, crucial for sensorless control, is primarily tasked with estimating the rotor’s position.

Conventional rotor flux observers in PMSM primarily analyze the interactions among stator current, voltage, and rotor flux to infer the rotor’s position and speed. This method involves two critical steps: estimating the flux and calculating the position-speed parameters. 

To simplify the derivation, this analysis excludes core saturation, eddy currents, and hysteresis losses. Additionally, it assumes no damping effects in either the rotor or the permanent magnet, considering the back electromotive force to be sinusoidal. As a result, within a stationary reference frame, the equations for voltage and flux in the PMSM are structured in the following manner:

(1) $$\left[\begin{array}{c} u_{\alpha }\\ u_{\beta } \end{array}\right]=\left[\begin{array}{cc} R_{s} & 0\\ 0 & R_{s} \end{array}\right]\left[\begin{array}{c} i_{\alpha }\\ i_{\beta } \end{array}\right]+p\left[\begin{array}{c} \Psi_{\alpha }\\ \Psi_{\beta } \end{array}\right]$$

(2) $$\left[\begin{array}{c} \Psi_{\alpha }\\ \Psi_{\beta } \end{array}\right]=L\cdot \left[\begin{array}{c} i_{\alpha }\\ i_{\beta } \end{array}\right]+\psi_{f}\cdot \left[\begin{array}{c} \cos\left(\theta_{e}\right)\\ \sin\left(\theta_{e}\right) \end{array}\right]$$

where $u_{s}={\left[\begin{array}{cc} u_{\alpha } & u_{\beta } \end{array}\right]}^{T}$ is the stator voltage vector, $R_{s}$ is the stator resistance, $i_{s}={\left[\begin{array}{cc} i_{\alpha } & i_{\beta } \end{array}\right]}^{T}$ is the stator current, p symbolizes the derivative (time derivative), $\Psi_{s}={\left[\begin{array}{cc} \Psi_{\alpha } & \Psi_{\beta } \end{array}\right]}^{T}$ is the stator flux vector, L is the inductance matrix, $\psi_{f}$ is the permanent magnet flux, and $\theta_{e}$ is the actual position of the rotor.

Differentiating the flux yields the back electromotive force, from which the state variables x and y are constructed. Here, $x={\left[\begin{array}{cc} x_{\alpha } & x_{\beta } \end{array}\right]}^{T}$ is the stator flux $\Psi_{s}$, $x-Li_{s}$ is the rotor flux vector $\Psi_{r}={\left[\begin{array}{cc} \Psi_{r\alpha } & \Psi_{r\beta } \end{array}\right]}^{T}$, and $y={\left[\begin{array}{cc} y_{\alpha } & y_{\beta } \end{array}\right]}^{T}$ is the back electromotive force vector.

(3) $$\left[\begin{array}{c} x_{\alpha }\\ x_{\beta } \end{array}\right]=L\cdot \left[\begin{array}{c} i_{\alpha }\\ i_{\beta } \end{array}\right]+\psi_{f}\cdot \left[\begin{array}{c} \cos\left(\theta_{e}\right)\\ \sin\left(\theta_{e}\right) \end{array}\right]$$

(4) $$\left[\begin{array}{c} y_{\alpha }\\ y_{\beta } \end{array}\right]=\left[\begin{array}{c} u_{\alpha }\\ u_{\beta } \end{array}\right]-\left[\begin{array}{cc} R_{s} & 0\\ 0 & R_{s} \end{array}\right]\left[\begin{array}{c} i_{\alpha }\\ i_{\beta } \end{array}\right]$$

From Equations (1) and (2), a state-space equation can be constructed:

(5) $$\dot{x}=y$$

To develop a nonlinear observer, a vector function η is defined.

(6) $$\eta \left(x\right)=x-L\cdot \left[\begin{array}{c} i_{\alpha }\\ i_{\beta } \end{array}\right]$$

The final nonlinear flux observer is represented as follows:

(7) $$\dot{\hat{x}}=y+\frac{\gamma }{2}\eta \left(\hat{x}\right)\left[\psi_{f}^{2}-{\left\|\eta \left(\hat{x}\right)\right\|}^{2}\right]$$

where $\hat{x}$ is the observer’s state variable, and γ is the observer’s gain coefficient.

Equation (3) reveals that the rotor flux vector holds essential positional information about the rotor, facilitating the computation of the rotor’s electrical angle, detailed as follows:

(8) $$\hat{\theta }=\tan^{-1}\left(\frac{\hat{x_{\beta }}-Li_{\beta }}{\hat{x_{\alpha }}-Li_{\alpha }}\right)=\tan^{-1}\left(\frac{{\hat{\Psi }}_{r\beta }}{{\hat{\Psi }}_{r\alpha }}\right)$$

Equation (8) demonstrates that errors in the estimation of the rotor flux vector significantly affect the accuracy of calculating the rotor’s electrical angle.

**Figure 2:**
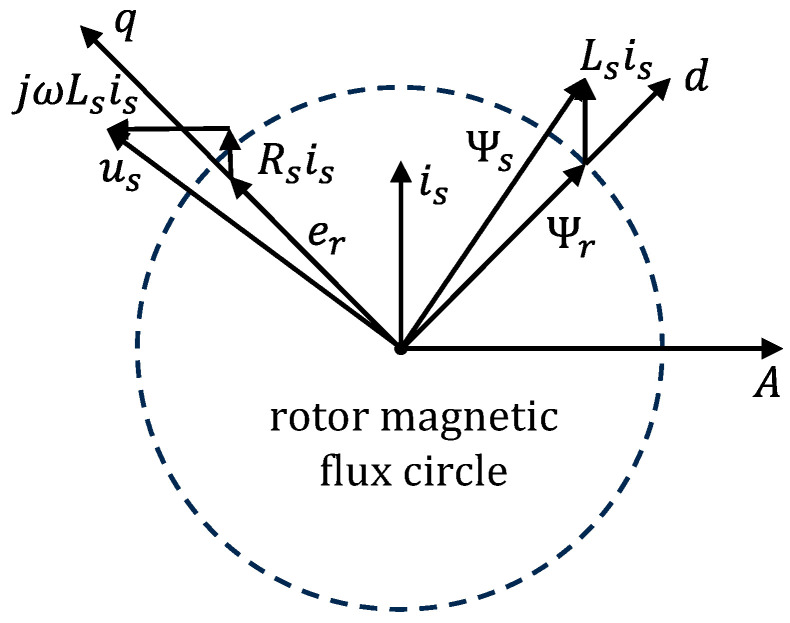
Vector diagram of rotor flux observation algorithm.

In order to visualize the flux observation algorithm, Figure 2 depicts the voltage equation (Equation (1)) and flux equation (Equation (2)) of the mathematical model of the permanent magnet synchronous motor in a vector way. The rotor flux circle $\Psi_{r}$ represented by the dotted line in the figure can be calculated through the mathematical model, and its vector direction, which is the rotor angle of the motor, can then be obtained. It can be seen that accurately estimating the flux information is the core of the flux observation algorithm, and will also directly affect the accurate output of the observer. Hence, it is crucial to develop an algorithm that accurately estimates the rotor flux, closely linked to the rotor’s position, to ensure stable and effective motor control. 

## 3. Flux Observer Based on SOGIFO-X

### 3.1. Error Analysis

Influenced by external environmental factors, the rotor flux vector incorporates a variety of disturbance signals, mainly composed of DC components and higher-order harmonics.

Based on Equations (1) and (2), the rotor flux can be expressed as the integral of the back electromotive force (EMF), leading to the following derivation:

(9) $$\Psi_{r}=\int \left(u_{s}-R_{s}i_{s}-Lpi_{s}\right)\;dt=\int e_{r}\;dt$$

where $e_{r}$ is the back EMF vector, with $e_{r}={\left[\begin{array}{cc} e_{r\alpha } & e_{r\beta } \end{array}\right]}^{T}$. The initial value for the integration of the estimated rotor flux vector is defined as $\Psi_{r}\left(0\right)=\Psi_{s}\left(0\right)-Li_{s}\left(0\right)$.

Changes in temperature and current can affect stator inductance and resistance, impacting the estimation of flux magnitude. Additionally, the drive system is prone to various disturbances, such as parameter mismatches, uncertain initial integration values, measurement errors, and converter non-linearities. This necessitates an adjustment to Equation (9), which is elaborated below:

(10) $$\Psi_{r}=\int \left(u_{s}-\left(R_{s0}+\Delta R_{s}\right)i_{s}-\left(L_{0}+\Delta L\right)pi_{s}+e_{r0}+\chi \right)dt$$

where $R_{s0}$ is the initial value of stator resistance, $\Delta R_{s}$ is the change in stator resistance, $L_{0}$ is the initial inductance value, ΔL indicates the change in inductance, $e_{r0}$ is the initial electromotive force, and χ is a compensation factor.

Equation (9) represents a stable value, known as the fundamental component, and contrasts with disturbances composed of DC and harmonic elements. Consequently, Equation (10) is revised to the following form:

(11) $$e_{r}=A_{0}+A_{1}\sin\left(\omega_{1}t+\varphi_{1}\right)+\sum A_{h}\sin\left(\omega_{h}t+\varphi_{h}\right)$$

where $A_{0}$, $A_{1}$, and $A_{h}$ are the amplitude coefficients of the DC component, fundamental component, and harmonic components, respectively. $\omega_{1}$ and $\omega_{h}$ are the angular frequencies of the fundamental and harmonic components, while $\varphi_{1}$ and $\varphi_{h}$ are their respective phase angles. The summation symbol ∑ is the cumulative addition of all harmonic components.

Upon applying the Laplace transform, the equation becomes:

(12) $$E_{r}\left(s\right)=\frac{A_{0}}{s}+\frac{A_{1}\left(s\sin\varphi_{1}+\omega_{1}\cos\varphi_{1}\right)}{s^{2}+\omega_{1}^{2}}+\sum \frac{A_{h}\left(s\sin\varphi_{h}+\omega_{h}\cos\varphi_{h}\right)}{s^{2}+\omega_{h}^{2}}$$

where $E_{r}\left(s\right)$ is the Laplace transform of $E_{r}$, and s is the Laplace operator.

### 3.2. Traditional Algorithm Analysis

The performance of the algorithm introduced in this paper is evaluated by comparing it with established methods like the pure integrator, LPF, and SOGIFO. 

#### 3.2.1. Integrator

Traditional flux observers utilize a first-order integrator, represented by 1/s in the flux estimation process. Considering the aforementioned nonlinear disturbances, the observed rotor flux $E_{r}\left(s\right)\cdot 1/s$, after undergoing inverse Laplace transformation, is expressed as:

(13) $$\begin{array}{c} \Psi_{r\_I}\left(t\right)=A_{0}t+\frac{A_{1}}{\omega_{1}}\cos\left(\varphi_{1}\right)+\sum_{h=2}^{\infty }\frac{A_{h}}{\omega_{h}}\cos\left(\varphi_{h}\right)+\frac{A_{1}}{\omega_{1}}\sin\left(\omega_{1}t+\varphi_{1}-0.5\pi t\right)\\ +\sum_{h=2}^{\infty }\frac{A_{1h}}{\omega_{h}}\sin\left(\omega_{h}t+\varphi_{h}-0.5\pi t\right) \end{array}$$

This expression comprises a DC bias component $A_{0}t+\frac{A_{1}}{\omega_{1}}\cos\left(\varphi_{1}\right)+\sum_{h=2}^{\infty }\frac{A_{h}}{\omega_{h}}\cos\left(\varphi_{h}\right)$, a fundamental component $\frac{A_{1}}{\omega_{1}}\sin\left(\omega_{1}t+\varphi_{1}-0.5\pi t\right)$, and higher-order harmonic components $\sum_{h=2}^{\infty }\frac{A_{1h}}{\omega_{h}}\sin\left(\omega_{h}t+\varphi_{h}-0.5\pi t\right)$. The term $A_{0}t$ suggests a linear increase in DC bias over time, resulting in a substantial offset in the integrator’s output, even with minimal DC components. This can greatly affect the integration outcomes, possibly causing considerable distortion from magnetic flux saturation. Such discrepancies can lead to errors in estimating rotor position, potentially compromising the feasibility of sensorless control in extreme cases. Additionally, the presence of harmonic elements in the rotor flux chain estimation may adversely affect the accuracy of position measurement.

#### 3.2.2. LPF

Digital low pass filters are frequently used to reduce magnetic saturation, DC bias, and higher-order harmonic interference. This method eliminates the DC drift component by subtracting it from the flux chain or applying a high-pass filter after integration. This process removes the DC component from the fundamental flux chain, akin to a low-pass filter’s effect. Choosing an appropriate cutoff frequency effectively minimizes DC bias and higher-order harmonics in the flux chain integration process. However, this method may result in some amplitude reduction and phase lag, necessitating the implementation of compensatory strategies.

Consider $E_{r}\left(s\right)\cdot 1/\left({s+\omega }_{c}\right)$, and after applying the inverse Laplace transform:

(14) $$\begin{array}{c} \Psi_{r\_LPF}\left(t\right)=\frac{A_{0}}{\omega_{c}}-\frac{A_{0}e^{-\omega_{c}t}}{\omega_{c}}+\frac{A_{1}\cos\left(\varphi_{1}+\theta_{1}\right)e^{-\omega_{c}t}}{\sqrt{\omega_{1}^{2}+\omega_{c}^{2}}}+\sum_{h=2}^{\infty }\frac{A_{h}\cos\left(\varphi_{h}+\theta_{h}\right)}{\sqrt{\omega_{h}^{2}+\omega_{c}^{2}}}\\ +\frac{A_{1}}{\sqrt{\omega_{1}^{2}+\omega_{c}^{2}}}\sin\left(\omega_{1}t+\varphi_{1}-0.5\pi t+\theta_{1}\right)\\ +\sum_{h=2}^{\infty }\frac{A_{h}}{\sqrt{\omega_{h}^{2}+\omega_{c}^{2}}}\sin\left(\omega_{h}t+\varphi_{h}-0.5\pi t+\theta_{h}\right) \end{array}$$

where $\frac{A_{0}}{\omega_{c}}-\frac{A_{0}e^{-\omega_{c}t}}{\omega_{c}}+\frac{A_{1}\cos\left(\varphi_{1}+\theta_{1}\right)e^{-\omega_{c}t}}{\sqrt{\omega_{1}^{2}+\omega_{c}^{2}}}+\sum_{h=2}^{\infty }\frac{A_{h}\cos\left(\varphi_{h}+\theta_{h}\right)}{\sqrt{\omega_{h}^{2}+\omega_{c}^{2}}}$ is the DC bias component, $\frac{A_{1}}{\sqrt{\omega_{1}^{2}+\omega_{c}^{2}}}\sin\left(\omega_{1}t+\varphi_{1}-0.5\pi t+\theta_{1}\right)$ is the fundamental component, and $\sum_{h=2}^{\infty }\frac{A_{h}}{\sqrt{\omega_{h}^{2}+\omega_{c}^{2}}}\sin\left(\omega_{h}t+\varphi_{h}-0.5\pi t+\theta_{h}\right)$ is the higher-order harmonic component. The DC component gradually decreases exponentially over time. Although it cannot be completely eliminated, this method effectively addresses magnetic saturation issues. Additionally, it significantly reduces the amplitude of higher-order harmonics. However, using a LPF also results in a reduction of the fundamental wave’s amplitude and introduces a phase delay. Compared to the integrator, the attenuation of the fundamental wave amplitude is $\frac{\omega_{1}}{\sqrt{\omega_{1}^{2}+\omega_{c}^{2}}}$, with a phase delay $\theta_{1}=\arctan\left(\frac{\omega_{c}}{\omega_{1}}\right)$. Therefore, compensation for amplitude and phase is required.

#### 3.2.3. SOGIFO

**Figure 3:**
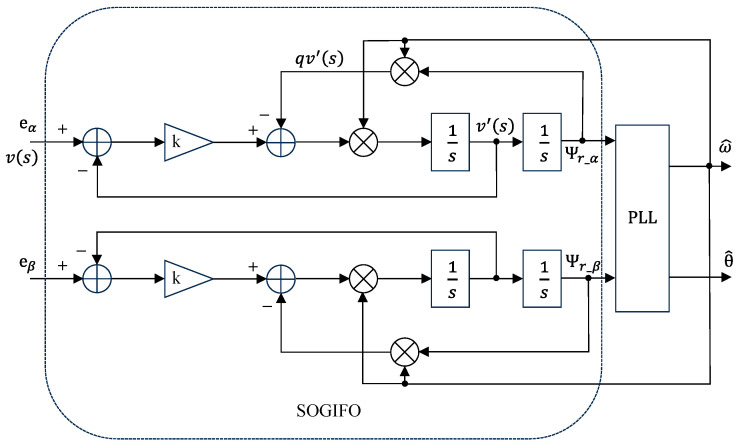
Block diagram of flux observer based on SOGIFO.

SOGI can generate two mutually orthogonal signals, and its structure is illustrated in Figure 3. v is the input signal. The outputs, $v^{\prime }$ and $qv^{\prime }$, are two orthogonal signals, with $v^{\prime }$ leading $qv^{\prime }$ by a phase of 90°. The transfer function of SOGI is given by:

(15) $$D\left(s\right)=\frac{v^{\prime }\left(s\right)}{v\left(s\right)}=\frac{k\omega^{\prime }s}{s^{2}+k\omega^{\prime }s+\omega^{\prime 2}}$$

(16) $$Q\left(s\right)=\frac{qv^{\prime }\left(s\right)}{v\left(s\right)}=\frac{k\omega^{\prime 2}}{s^{2}+k\omega^{\prime }s+\omega^{\prime 2}}$$

where v is the input sinusoidal voltage signal, and k is the damping coefficient. 

When the filter’s center frequency $\omega^{\prime }$ matches the input voltage signal’s frequency ω, the output signals $v^{\prime }$ and $qv^{\prime }$ become sinusoidal waves with equal amplitude and a 90° phase difference. $v^{\prime }$ shares the same phase and amplitude as v. D(s) is commonly used for filtering, and Q(s) serves the purpose of integration.

Applying the SOGI in flux observers involves using the back EMF vector as the input to SOGI. The structure of the Second-Order Generalized Integrative Flux Observer (SOGIFO) is shown in Figure 3.

(17) $$\Psi_{r\_SOGIFO}\left(s\right)=\frac{1}{\omega^{\prime }}Q\left(s\right)\cdot E_{r}\left(s\right)=\frac{k\omega^{\prime }}{s^{2}+k\omega^{\prime }s+\omega^{\prime 2}}E_{r}\left(s\right)=\frac{1}{s}E_{r}\left(s\right)$$

Equation (17) demonstrates that using SOGIFO can achieve the same effect as the integrator component in traditional flux observers.

Consider $E_{r}\left(s\right)\cdot \frac{k\omega^{\prime }}{s^{2}+k\omega^{\prime }s+\omega^{\prime 2}}$, and after applying the inverse Laplace transform:

(18) $$\begin{array}{c} \Psi_{r\_SOGIFO}\left(t\right)=\frac{A_{0}k}{\omega_{1}}+\frac{A_{1}}{\omega_{1}}\sin\left(\omega_{1}t+\varphi_{1}-0.5\pi t\right)+\sum_{h=2}^{\infty }\frac{A_{h}}{\omega_{h}}\\ \cdot \frac{1}{\sqrt{{\left(1-h^{2}\right)}^{2}/k^{2}h^{2}+1}}\cdot \sin\left(\omega_{h}t+\varphi_{h}-0.5\pi t+\gamma_{h1}\right) \end{array}$$

where $\frac{A_{0}k}{\omega_{1}}$ is the DC bias component, $\frac{A_{1}}{\omega_{1}}\sin\left(\omega_{1}t+\varphi_{1}-0.5\pi t\right)$ is the fundamental component, and $\sum_{h=2}^{\infty }\frac{A_{h}}{\omega_{h}}\cdot \frac{1}{\sqrt{{\left(1-h^{2}\right)}^{2}/k^{2}h^{2}+1}}\cdot \sin\left(\omega_{h}t+\varphi_{h}-0.5\pi t+\gamma_{h1}\right)$ is the higher-order harmonic component. It is observed that, compared to a pure integrator, the fundamental component does not exhibit any change in amplitude and phase, and the higher-order harmonic components are significantly reduced. The DC bias component is attenuated, addressing the issue of magnetic saturation, but it is not completely eliminated.

### 3.3. SOGIFO-X

**Figure 4:**
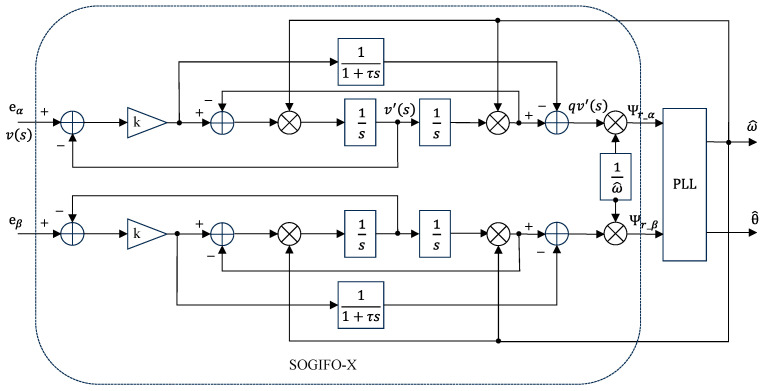
Block diagram of flux observer based on SOGIFO-X.

An enhanced SOGIFO-X technique has been introduced, designed to surmount the challenges in DC bias elimination inherent in traditional SOGIFO. This approach focuses on refined flux filtering and integration for improved performance. Illustrated in Figure 4, this sophisticated method incorporates a low-pass filter into the SOGIFO framework, efficiently minimizing higher-order harmonic interference and substantially reducing $qv^{\prime }$ in the high-frequency range. Consequently, SOGIFO-X excels in simultaneously mitigating both the DC component and higher-order harmonics within the input signal. The transfer function for SOGIFO-X is delineated below:

(19) $$D\left(s\right)=\frac{v^{\prime }\left(s\right)}{v\left(s\right)}=\frac{k\omega^{\prime }s}{s^{2}+k\omega^{\prime }s+\omega^{\prime 2}}$$

(20) $$Q\left(s\right)=\frac{qv^{\prime }\left(s\right)}{v\left(s\right)}=\frac{k\left(\tau \omega^{\prime 2}s-s^{2}\right)}{\left(s^{2}+k\omega^{\prime }s+\omega^{\prime 2}\right)\left(1+\tau s\right)}$$

where k is the damping coefficient, and τ is the time constant of the low pass filter.

Similar to SOGI, $D\left(s\right)$ is typically used for filtering, and $Q\left(s\right)$ serves the role of integration. $qv^{\prime }$ can be considered the integrated flux of the input back EMF, defined at steady state as s=jω. The calculation formula is:

(21) $$\begin{array}{c} \;\;\;\;\Psi_{r\_SOGIFO-X}\left(s\right)=\frac{1}{\omega^{\prime }}Q_{SOGIFO-X}\left(s\right)\cdot E_{r}\left(s\right)\\ =\frac{1}{\omega^{\prime }}\frac{k\left(\tau \omega^{\prime 2}s-s^{2}\right)}{\left(s^{2}+k\omega^{\prime }s+\omega^{\prime 2}\right)\left(1+\tau s\right)}E_{r}\left(s\right)\\ =\frac{1}{\omega^{\prime }}\frac{k\left(\tau \omega^{\prime 2}s+\omega^{\prime 2}\right)}{k\omega^{\prime }s\cdot \left(1+\tau s\right)}E_{r}\left(s\right)=\frac{1}{s}E_{r}\left(s\right) \end{array}$$

Equation (21) demonstrates that the improved SOGIFO-X can be used to estimate the motor rotor flux vector information.

Consider $E_{r}\left(s\right)\cdot \frac{1}{\omega^{\prime }}\frac{k\left(\tau \omega^{\prime 2}s-s^{2}\right)}{\left(s^{2}+k\omega^{\prime }s+\omega^{\prime 2}\right)\left(1+\tau s\right)}$, and after applying the inverse Laplace transform:

- DC Component

Based on the final value theorem of Laplace transform, we obtain:

(22) $$\begin{array}{c} \;\;\;\;\underset{t\to \infty }{\lim}\Psi_{SOGIFO-X}\left(t\right)=\underset{s\to 0}{\lim}s\Psi_{SOGIFO-X}\left(s\right)\\ =\underset{s\to 0}{\lim}s\frac{A_{0}}{s}\frac{1}{\omega^{\prime }}\frac{k\left(\tau \omega^{\prime 2}s-s^{2}\right)}{\left(s^{2}+k\omega^{\prime }s+\omega^{\prime 2}\right)\left(1+\tau s\right)}=0 \end{array}$$

- Fundamental Component

Substituting s=jω into Equation (21), we obtain:

(23) $$\Psi_{r\_SOGIFO-X}\left(s\right)=\frac{1}{j\omega }E_{r}\left(s\right)=\frac{A_{1}}{\omega_{1}}\sin\left(\omega_{1}t+\varphi_{1}-0.5\pi \right)$$

- Higher-Order Harmonics

With τ as the time constant of the LPF, taking $\tau =\frac{2\pi }{\omega }$, and substituting $s=j\omega_{h}=jh\omega$ into Equation (21), we obtain:

(24) $$\begin{array}{c} \frac{1}{\omega^{\prime }}\frac{k\left(\tau \omega^{\prime 2}s-s^{2}\right)}{\left(s^{2}+k\omega^{\prime }s+\omega^{\prime 2}\right)\left(1+\tau s\right)}\\ =\frac{kh}{\omega }\frac{\sqrt{h^{2}+4\pi^{2}}}{\sqrt{{\left(1-h^{2}-2\pi Kh^{2}\right)}^{2}+{\left(kh+2\pi h-2\pi h^{3}\right)}^{2}}}∠\theta \end{array}$$

where:

(25) $$\theta =\tan^{-1}\left(\frac{2\pi }{h}\right)-\tan^{-1}\left(\frac{kh+2\pi h-2\pi h^{3}}{1-h^{2}-2\pi kh^{2}}\right)$$

By incorporating the harmonic components into Equation (21), the higher-order harmonics of the flux can be represented as:

(26) $$\begin{array}{c} \sum_{h=2}^{\infty }\frac{A_{h}}{\omega_{h}}\cdot \frac{1}{\sqrt{\frac{{\left(1-h^{2}-2\pi kh^{2}\right)}^{2}+{\left(kh+2\pi h-2\pi h^{3}\right)}^{2}}{k^{2}h^{4}\left(h^{2}+4\pi^{2}\right)}}}\\ \cdot \sin\left(\omega_{h}t+\varphi_{h}-0.5\pi t+\gamma_{h2}\right) \end{array}$$

where:

(27) $$\theta =\tan^{-1}\left(\frac{kh+2\pi h-2\pi h^{3}}{1-h^{2}-2\pi kh^{2}}\right)-\tan^{-1}\left(\frac{2\pi }{h}\right)$$

In summary, the flux chain after filtering integration by using the SOGIFO-X is as follows:

(28) $$\begin{array}{c} \Psi_{r\_SOGIFO-X}\left(t\right)=\frac{A_{1}}{\omega_{1}}\sin\left(\omega_{1}t+\varphi_{1}-0.5\pi \right)+\sum_{h=2}^{\infty }\frac{A_{h}}{\omega_{h}}\\ \cdot \frac{1}{\sqrt{\frac{{\left(1-h^{2}-2\pi kh^{2}\right)}^{2}+{\left(kh+2\pi h-2\pi h^{3}\right)}^{2}}{k^{2}h^{4}\left(h^{2}+4\pi^{2}\right)}}}\\ \cdot \sin\left(\omega_{h}t+\varphi_{h}-0.5\pi t+\gamma_{h2}\right) \end{array}$$

The Is derived from the specified equation demonstrate that SOGIFO-X adeptly eliminates the DC component within the input signal and effectively attenuates high-frequency elements. Additionally, at the fundamental frequency, the integration of the LPF does not incur any delay, thereby enabling the precise filtering of interference signals amidst flux chain integration.

**Figure 5:**
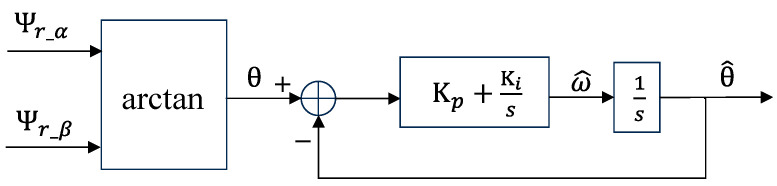
Internal structure of PLL.

Fluctuations in the control system’s quality factor, triggered by varying input signal frequencies, significantly influence its dynamic behavior. To address this, incorporating the phase angle $\omega_{0}$ into SOGIFO-X endows the flux observer with enhanced frequency responsiveness. This integration guarantees precise synchronization of SOGIFO-X with the input signal’s frequency, even amid variations in the rotor position signal’s frequency. The execution of this frequency adaptation is efficiently actualized using a PLL, as illustrated in Figure 5.

### 3.4. Bode Diagram Analysis

**Figure 6:**
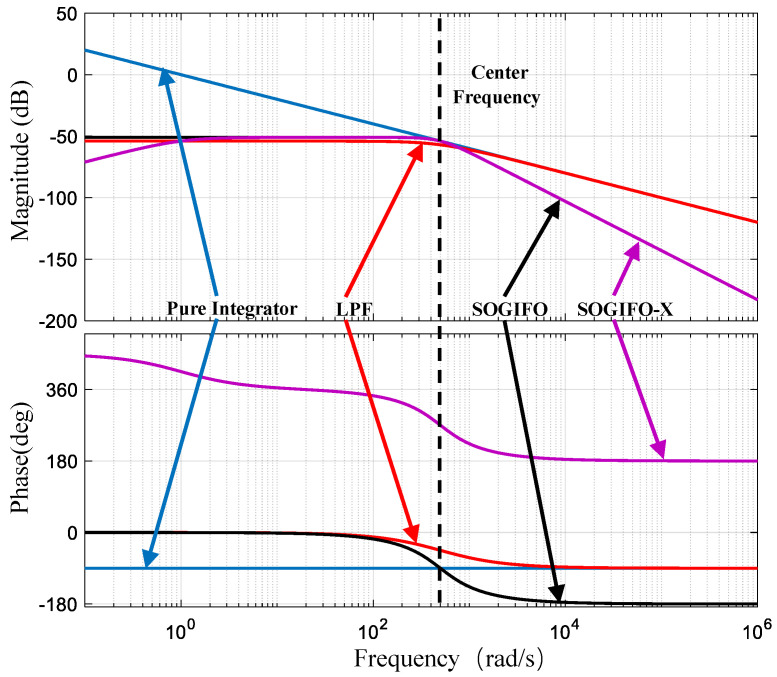
Bode diagrams of first-order integrator, LPF, SOGIFO, and SOGIFO-X. The blue, red, black, and purple lines represent the Bode plots of the first-order integral, LPF, SOGIFO, and SOGIFO-X, respectively.

The effectiveness of the SOGIFO-X method has been substantiated through rigorous mathematical analysis. Figure 6 shows the comparative Bode plot analysis of different algorithms.

The magnitude response of the first-order integrator shows a 20 dB/decade increase at lower frequencies. This indicates an amplified DC bias in flux deviation, which could lead to magnetic flux saturation.

Conversely, a LPF effectively diminishes the DC component, exhibiting a −20 dB/decade reduction in high frequencies, similar to the attenuation of a first-order integrator in this range. However, the LPF’s performance at the center frequency shows notable amplitude reduction and phase delay, suggesting the need for further tuning. 

SOGIFO’s low-frequency response parallels that of the LPF, implying a reduction, but not complete removal, of DC bias. Its high-frequency response, decreasing at −40 dB/decade, indicates a superior capacity to filter higher-order harmonics compared to the LPF. At the center frequency, SOGIFO maintains consistency with a first-order integrator, preserving the integrity of the fundamental wave. 

SOGIFO-X excels in minimizing both DC components and harmonics, with its low-frequency response reducing at −20 dB/decade, signifying complete DC bias removal. At high frequencies, its −40 dB/decade reduction mirrors SOGIFO’s high-order harmonic filtering capability, while keeping the fundamental wave unaffected. 

In summary, SOGIFO-X demonstrates superior filtering effectiveness, as elaborated on in Table 1.

## 4. Simulation and Experiment

### 4.1. Simulation Analysis

A control system for a PMSM was constructed and tested using Simulink, serving to confirm the efficacy of the control approach suggested in this study. The performance of flux observers based on pure integrators, LPF, SOGIFO, and SOGIFO-X is analyzed and compared.

The parameters of the selected PMSM are as outlined in Table 2.

#### 4.1.1. Steady-State Performance of the Algorithm

The four algorithms were tested in the same operating conditions to compare their flux estimation accuracy. This comparison highlighted each algorithm’s ability to reduce DC bias and high-order harmonics. Furthermore, by analyzing the differences between the estimated and actual rotor positions, the effectiveness of the rotor position state observer in each algorithm was assessed. This approach ensures a fair and comprehensive evaluation of the algorithms’ performance in practical settings.

**Figure 7:**
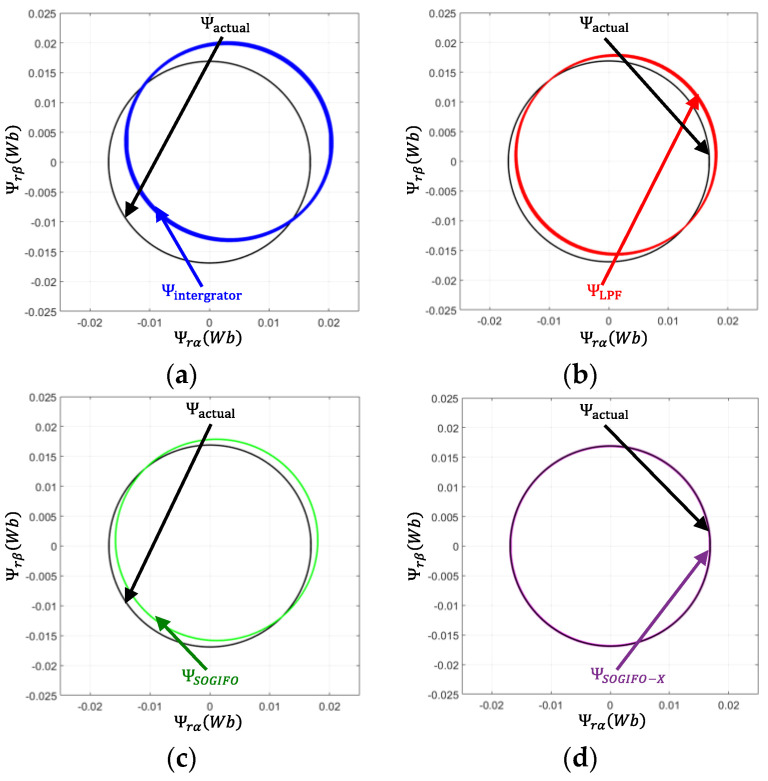
Rotor flux vector at 1200 rpm. (**a**) Integrator. (**b**) LPF. (**c**) SOGIFO. (**d**) SOGIFO-X. The black line represents the ideal magnetic flux circle of the rotor. The blue, red, green, and purple lines respectively represent the estimated rotor magnetic flux circles of Integral, LPF, SOGIFO, and SOGIFO-X.

Figure 7 presents a comparative analysis of the estimated α and β axis flux versus the ideal flux in a PMSM, employing various flux observers at a constant no-load speed of 1200 rpm. The ideal flux is represented as a black circle in this figure. The deviation of the actual flux center from the coordinate origin is indicative of the DC bias filtering effect, while the thickness of the solid line represents the effectiveness in filtering higher harmonics. By analyzing both aspects, namely DC bias and high-order harmonic filtering, the flux estimation capabilities of each algorithm can be comprehensively compared. The flux estimation by using the pure integrator exhibits a marked deviation from the ideal, indicating a significant DC bias effect. This variation highlights the limitations of the pure integrator in mitigating DC bias and high-frequency disturbances in flux estimation. Conversely, the flux circle estimated by using the compensated LPF shows less deviation, but still struggles with high-frequency disturbance filtration. SOGIFO demonstrates enhanced filtering of high frequencies, yet exhibits some offset. In stark contrast, SOGIFO-X successfully eliminates this offset, showcasing its exceptional accuracy in tracking the ideal flux.

**Figure 8:**
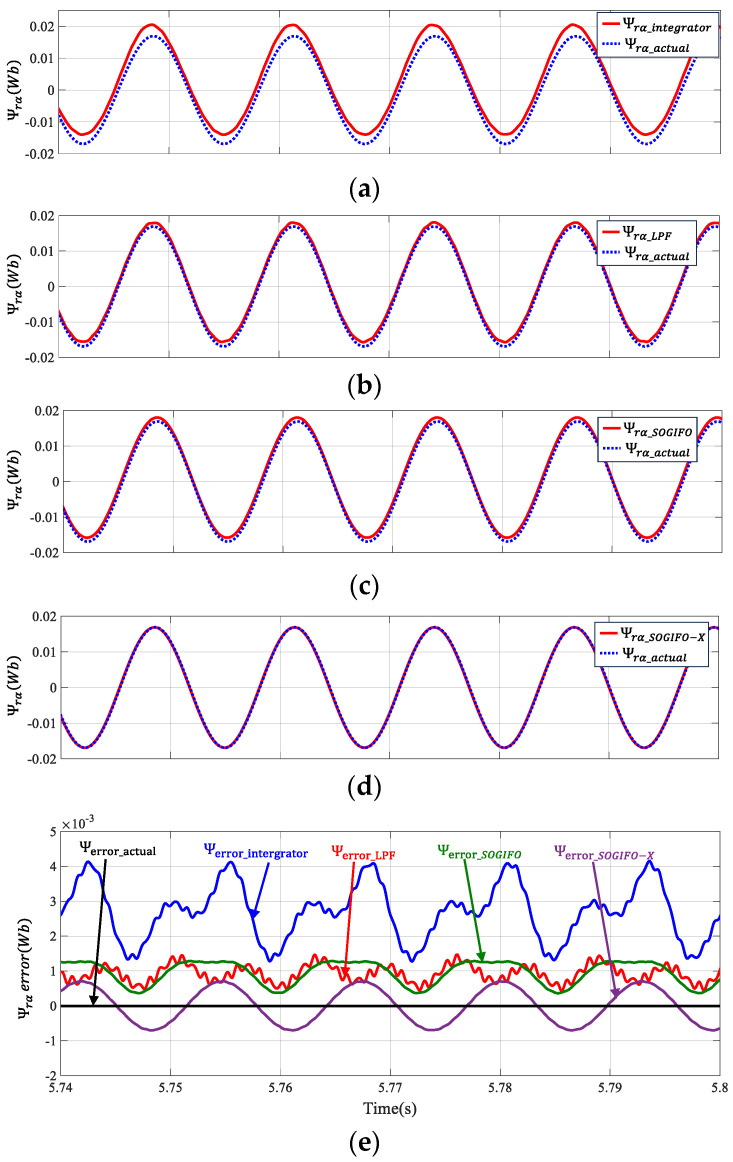
Flux vector estimation of α-axis rotor at 1200 rpm. (**a**) Integrator. (**b**) LPF. (**c**) SOGIFO. (**d**) SOGIFO-X. (**e**) Comparison of flux errors of four algorithms. The black line represents the ideal observed error of the rotor flux. The blue, red, green, and purple lines represent the observed errors of the Integral, LPF, SOGIFO and SOGIFO-X respectively for the rotor flux.

Figure 8 illustrates the α-axis flux estimation errors of four algorithms compared to the ideal flux. The deviation in flux along the X-axis is indicative of a DC offset error. Furthermore, the waveform’s sinuosity is a representation of the presence of higher harmonics. Table 3 shows the data analysis of the corresponding four algorithms. The mean error (ME) comparison reveals a markedly lower error for the SOGIFO-X algorithm compared to its three counterparts. In contrast to the consistently positive errors seen in other algorithms, SOGIFO-X’s errors fluctuate around zero, with a median close to zero, suggesting an effective elimination of DC bias. Additionally, the root mean square error (RMSE) for SOGIFO-X is substantially smoother than the others, indicating a more uniform error variation. Moreover, SOGIFO-X and SOGIFO exhibit significantly reduced high-frequency disturbances in their error patterns, underscoring their enhanced effectiveness in filtering higher-order harmonics.

**Figure 9:**
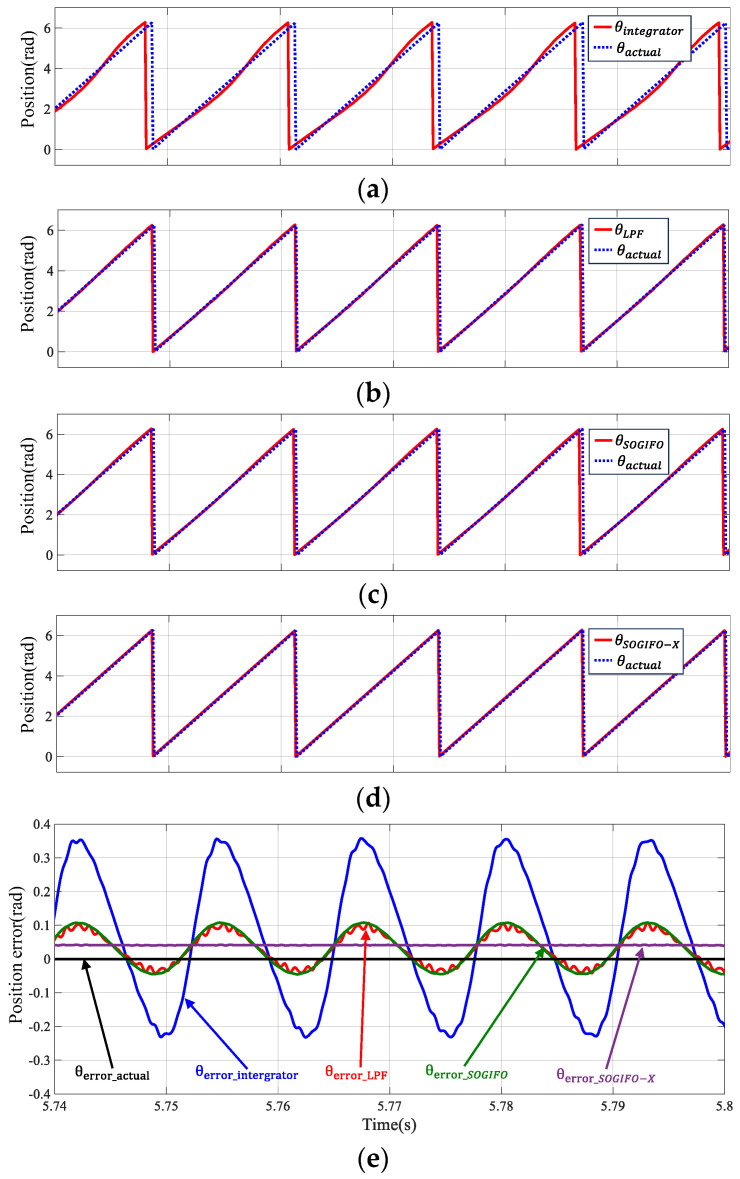
Rotor position estimation at 1200 rpm. (**a**) Integrator. (**b**) LPF. (**c**) SOGIFO. (**d**) SOGIFO-X. (**e**) Comparison of rotor position errors of four algorithms. The black line represents the ideal observed error of the rotor position. The blue, red, green, and purple lines represent the observed errors of the Integral, LPF, SOGIFO and SOGIFO-X respectively for the rotor position.

Figure 9 showcases the waveforms of rotor angle estimations amid DC bias and high-order harmonic interferences, analyzed using flux observers with four different algorithms. Table 4 shows the corresponding data analysis of the algorithm. The waveform analysis reveals that the rotor position error, as estimated using SOGIFO-X, is notably smaller compared to the other three algorithms. The standard deviation associated with SOGIFO-X is notably lower compared to the other three algorithms, indicating a more consistent and smoother estimated angle error. This underscores a substantial enhancement in rotor angle estimation accuracy achieved by using the algorithm introduced in this study, critically contributing to the motor’s stable operation.

**Figure 10:**
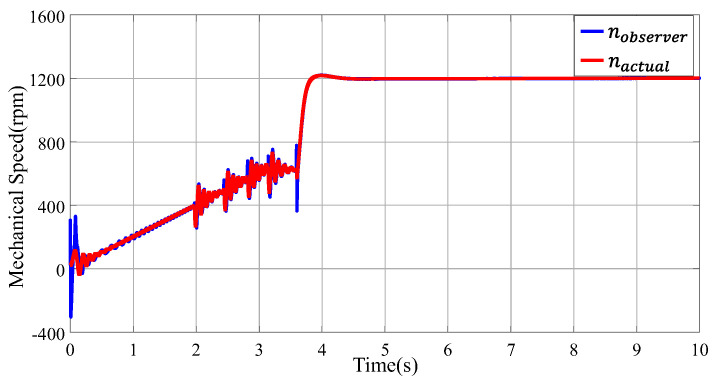
Velocity estimation in the full speed domain based on SOGIFO-X.

The flux observer’s ability to accurately determine rotor position is heavily reliant on the precision of flux estimation. At low speeds, factors like signal-to-noise ratio and dead zones, which introduce nonlinearity, can diminish the accuracy of flux estimation. This reduction in accuracy, in turn, leads to increased errors in rotor position determination, highlighting the challenge of maintaining precise control in these conditions. The accuracy of rotor position calculation by using the flux observer relies heavily on the flux estimation, which tends to be less accurate at lower speeds, leading to increased errors in rotor position determination under these conditions. To ensure a stable startup in low-speed scenarios, motors often employ I/F control mode. This involves setting an appropriate current-to-frequency ratio, allowing the motor to operate with open-loop speed control and closed-loop current control. In this setup, rotor position information is manually inputted, facilitating smoother and more reliable motor initiation at lower speeds. In the experimental framework, the system initially operates under Inverter Feed (I/F) mode for speeds up to 600 rpm, then gradually shifts to using the flux observer. Figure 10 shows a side-by-side comparison of rotor speeds as estimated by using the SOGIFO-X flux observer and the actual speeds. Remarkably, following the switch to the flux observer at 3.5 s, the estimated rotor speeds align closely with the actual speeds. The analysis indicates that the SOGIFO-X-based flux observer demonstrates high accuracy in estimating motor speed, particularly in the mid- and high-speed ranges.

#### 4.1.2. Dynamic Performance of the Algorithm

**Figure 11:**
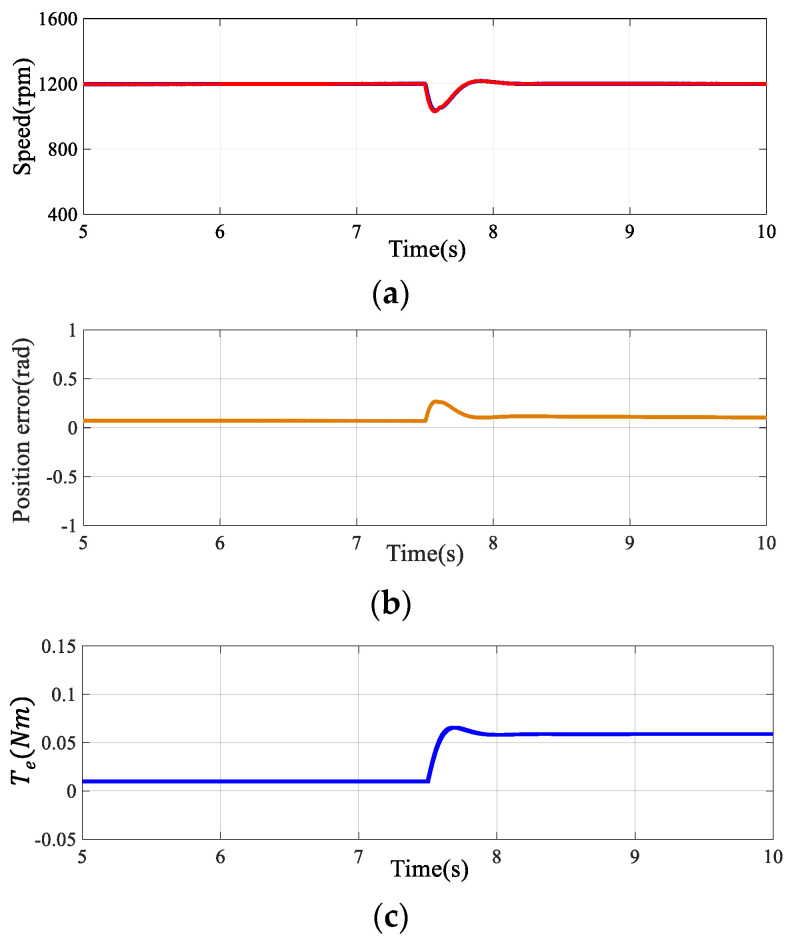

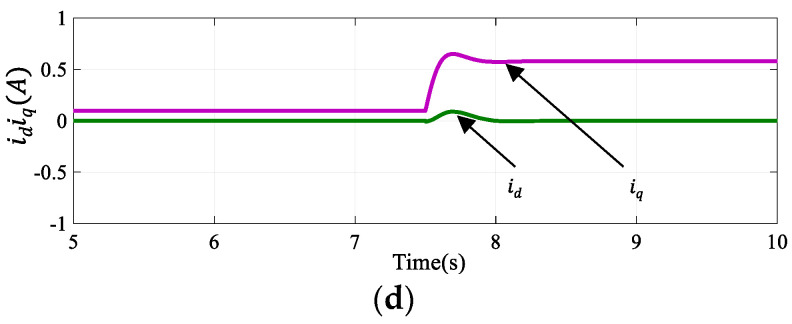
Dynamic response of the motor under 0.05 Nm torque perturbation applied at 7.5 s. (**a**) Speed response. (**b**) Rotor position estimation error. (**c**) Electromagnetic torque response. (**d**) D-axis and Q-axis current response. The green line represents the D-axis current and the purple line represents the Q-axis current in subfigure (**d**).

**Figure 12:**
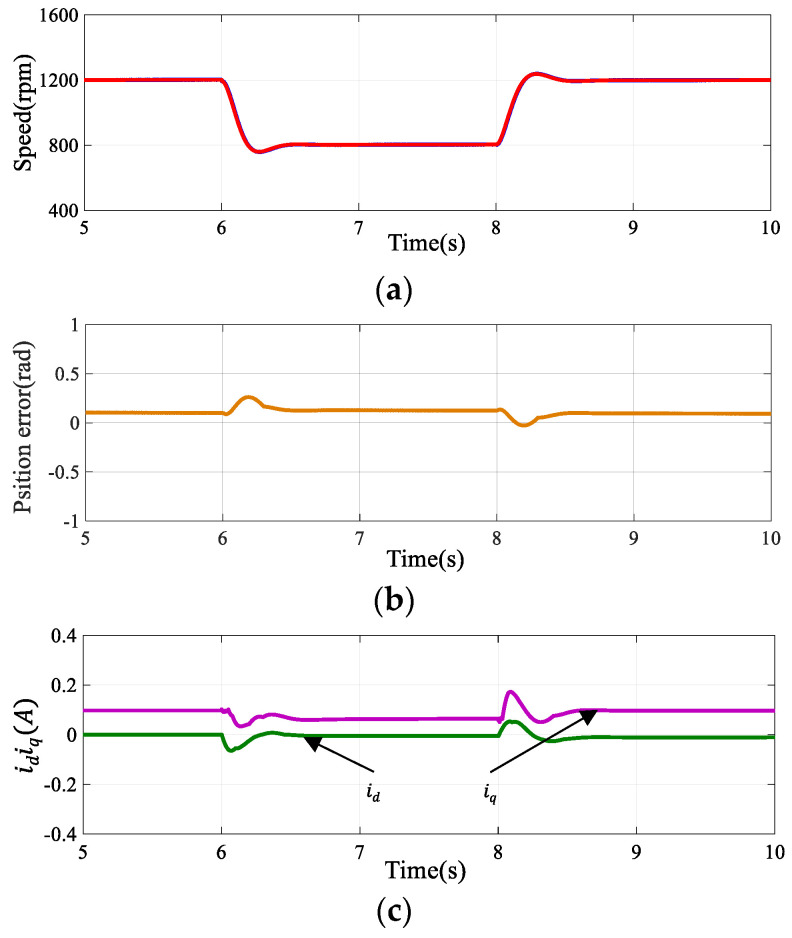
The reference speed changes from 1200 rpm to 800 rpm at 6 s and returns to 1200 rpm at 8 s. Simulation of motor dynamic response under this speed change. (**a**) Speed response. (**b**) Rotor position estimation error. (**c**) D-axis and Q-axis current response. The green line represents the D-axis current and the purple line represents the Q-axis current in (**c**).

The effectiveness of the proposed method was further substantiated by assessing the motor’s dynamic response to speed and torque variations using the SOGIFO-X-based flux observer. Figure 11 shows the motor’s performance when subjected to a 0.05 Nm load at 7.5 s, exhibiting response curves for speed, rotor error, electromagnetic torque, and phase current upon this sudden load change. Figure 12 illustrates the motor’s response to a rapid shift in reference speed: initially decreasing from 1200 rpm to 800 rpm at the 6-s mark, followed by a return to 1200 rpm after 8 s. It can be seen that in the case of disturbance, the individual waveforms quickly return to the stable state. In tandem, the estimated speed closely matches the actual speed, confirming SOGIFO-X’s precision in rotor position estimation. The rapid stabilization of phase current in response to load changes further attests to the algorithm’s dynamic adaptability.

Table 5 conducts a comprehensive analysis of the dynamic response within the scenarios depicted in Figure 11 and Figure 12, assessing multiple parameters, including overshoot, settling time, peak time, and rise time, with the motor’s speed response serving as the focal point. The empirical data indicates that—when confronted with abrupt changes in reference speed and load—the system is capable of swiftly reverting to a stable state. This evidences the system’s commendable dynamic capabilities.

### 4.2. Analysis of Experiment

**Figure 13:**
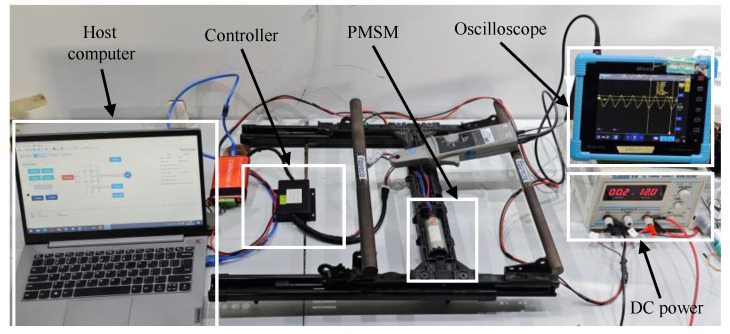
PMSM experimental platform.

In addition to simulations, practical tests of the SOGIFO-X were conducted on a dedicated PMSM testing platform, as illustrated in Figure 13. The tests utilized an STM32G4 board configured with a sampling frequency of 10 kHz for both FOC and sensorless algorithms. Initially, the motor accelerates to 600 rpm using I/F control in the zero-low speed range. To ensure a smooth start, the I/F mode employs a steady frequency increase for the position information. Once the motor achieves stable operation at medium and high speeds, it transitions to a flux observer mode for speed closed-loop control. In this phase, the rotor position information is entirely provided by the flux observer, facilitating seamless motor control across different speed ranges. Waveform data, recorded via an oscilloscope and serial port, reflected the motor’s operational parameters, which were consistent with those in the simulations.

**Figure 14:**
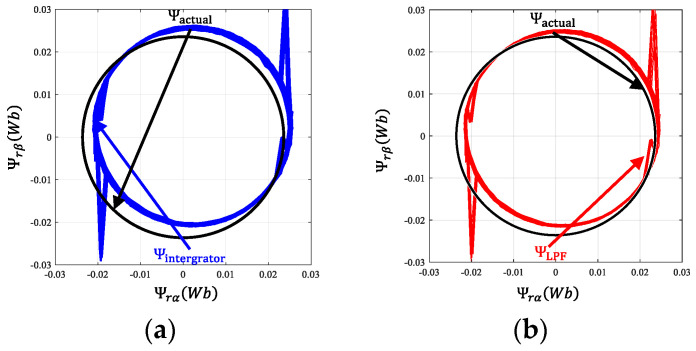

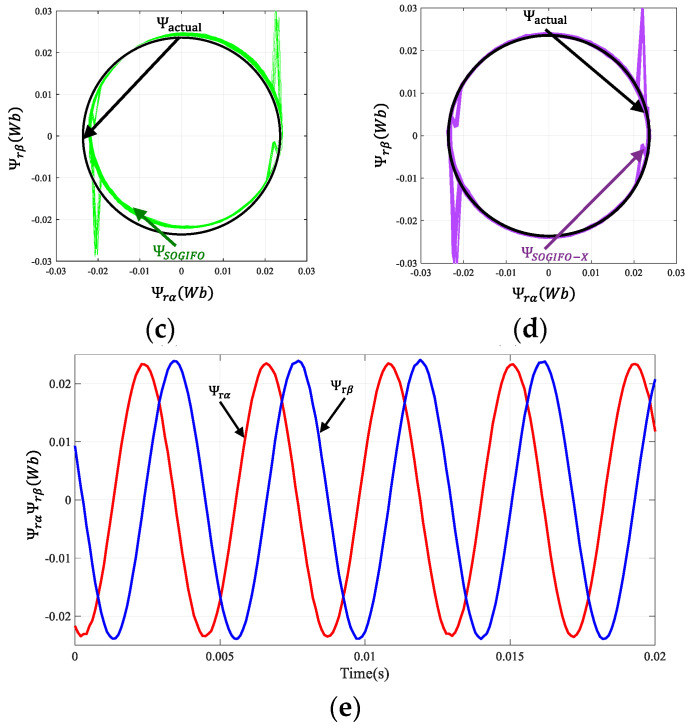
Rotor flux vector at 1200 rpm. (**a**) Integrator. (**b**) LPF. (**c**) SOGIFO. (**d**) SOGIFO-X. (**e**) Estimation of the αβ axis flux.

**Figure 15:**
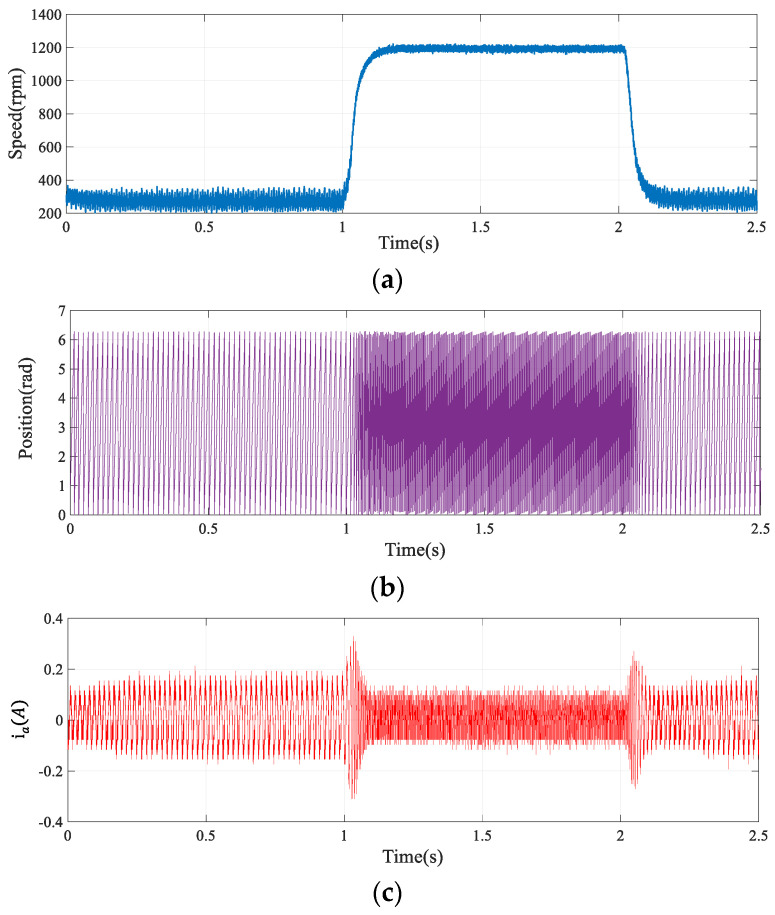
The reference speed changes from 1200 rpm to 300 rpm at 1 s, and returns to 1200 rpm at 2 s. Experiment of motor dynamic response under this speed variation. (**a**) Speed response. (**b**) Rotor position estimation. (**c**) Phase A current response.

**Figure 16:**
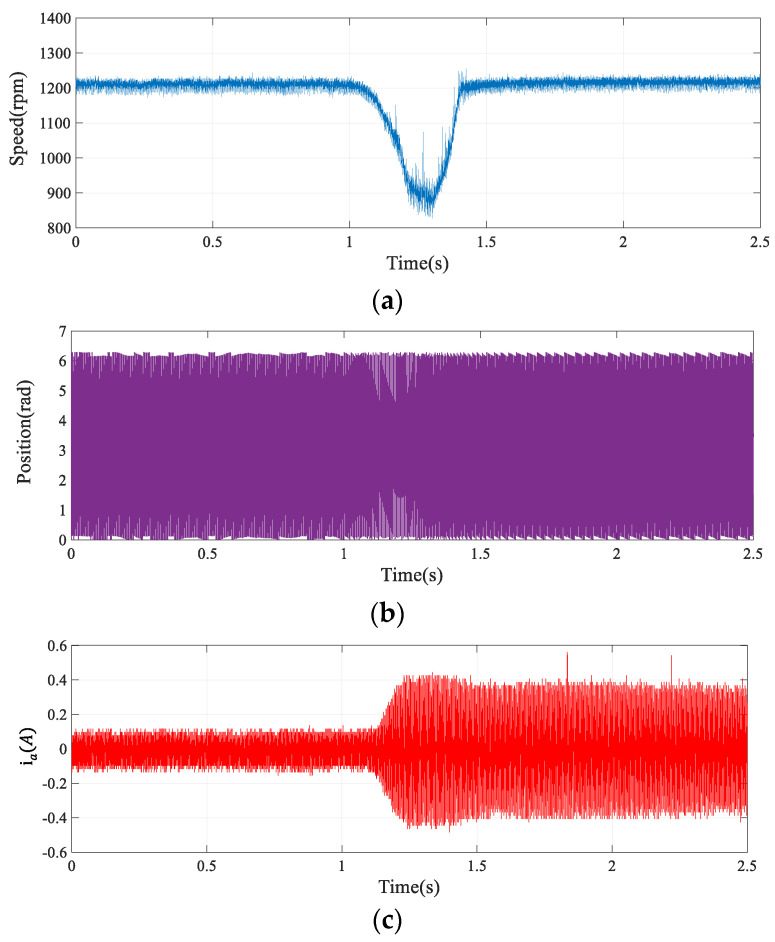
Dynamic response of the motor under 0.03 Nm load applied by 1 s (**a**) Speed response. (**b**) Rotor position estimation. (**c**) Phase A current response.

Figure 14 illustrates the motor’s estimated flux chain waveform at a steady speed of 1200 rpm, contrasting it with the ideal waveform, represented in black. The implementation of the advanced SOGIFO-X algorithm significantly refines the accuracy of flux chain estimation, harmonizing the experimental findings with simulation data. Figure 15 depicts the motor’s current and positional changes during the speed transition from 300 to 1200 rpm, showing the motor’s rapid return to stability post disturbances. Figure 16 shows the dynamic response of the motor under a 0.03 Nm load disturbance for 1 s, and the results show that the motor can quickly return to the stable state. The experiments demonstrate that the system aligns with the simulations, exhibiting robust dynamic performance.

The experimental findings confirm that the method markedly diminishes positional estimation error fluctuations, yields enhanced rotor position accuracy, and upholds robust dynamic performance.

## 5. Conclusions

The SOGIFO-X flux observer is presented as an innovative approach, designed to overcome the limitations of traditional flux observers in the sensorless control of PMSM. The proposed algorithm significantly reduces the high-order harmonics without changing the fundamental wave amplitude and phase, and completely eliminates the DC bias problem prevalent in the existing methods. Rigorous mathematical validation and Bode plot analysis, coupled with simulation and experimental verifications, confirm SOGIFO-X’s enhanced accuracy in flux linkage estimation. This leads to more precise rotor position determination and improved PMSM operational efficiency. Furthermore, SOGIFO-X demonstrates a robust dynamic response, broadening its practical application range. However, as its optimal performance is primarily observed in high-speed scenarios, future research should aim to adapt this algorithm for effective use in low-speed conditions.

## Figures and Tables

**Table 1:** Comparison of flux observer algorithms.

Algorithm	DC Bias	Higher-Order Harmonics	Flux Saturation	Fundamental Amplitude	Fundamental Phase
Integrator	High	Mid	Own	Undamped	No lag
LPF	Low	Mid	None	Damped	Lag
SOGIFO	Low	Low	None	Undamped	No lag
SOGIFO-X	None	Low	None	Undamped	No lag

**Table 2:** Main parameters of PMSM.

Symbol	Parameter	Value and Unit
$\psi_{f}$	Flux linkage of permanent magnet	0.0169 Wb
$P_{n}$	Number of pole pairs	4
$R_{s}$	Stator resistance	6.97 Ω
$L_{s}$	Stator inductance	5.35 mH
$N_{n}$	Rated speed	1250 rpm
J	Rotational inertia	$7.5\;*\;10^{-5}\;kg\cdot m^{2}$
$P_{n}$	Number of pole pairs	4

**Table 3:** Error analysis of α-axis rotor flux vector at 1200 rpm.

Algorithm	Sample Size	MAX	MIN	Midrange	ME	MAE	SD	RMSE
Integrator	6000	0.00415	0.00128	0.00272	0.00270	0.00270	0.00080	0.00281
LPF	6000	0.00147	0.00042	0.00095	0.00095	0.00095	0.00030	0.00101
SOGIFO	6000	0.00129	0.00036	0.00083	0.00092	0.00092	0.00024	0.00095
SOGIFO-X	6000	0.00071	−0.00070	0.000003	0.000015	0.00045	0.00035	0.00045

**Table 4:** Error analysis of rotor position at 1200 rpm.

Algorithm	Sample Size	MAX	MIN	Midrange	MAE	SD	RMSE
Integrator	6000	0.35764	−0.23238	0.06263	0.18696	0.20136	0.21293
LPF	6000	0.10867	−0.04346	0.03260	0.05313	0.04659	0.04691
SOGIFO	6000	0.10786	−0.04545	0.03120	0.04691	0.05380	0.05313
SOGIFO-X	6000	0.04252	0.03955	0.04104	0.04102	0.00051	0.04102

**Table 5:** Analysis of velocity response under different disturbances.

Condition	Steady-State Value	Peak Value	Overshoot	Peak Time	Transient Time	Rise Time
Variable load	1200	1217	1.4%	0.41	0.26	0.24
Variable speed	1200	1236	3%	0.28	0.37	0.14

## Data Availability

Data are contained within the article.
